# 

**Published:** 1881-05

**Authors:** 


### CONTENTS:

ART. I.—Neuralgia—Its Pathology and Etiology. By S. S. Wilson, M. D..............1
ART. II.—Syphilis About the Mouth. By F. N. Pedley, D D. S..............6
ART. III.—Odontological Society of Pennsylvania..............13
ART. IV.—Relative Action of Anæsthetics..............18
ART. V.—A Few Words about the Plastic Materials. By Geo. A. Mills, D. D. S..... 23
ART. VI.—Cohesive or Mechanical Force. By T. D. Shumway, D. D S..............26
ART. VII.—Correspondence—"Cohesion of Molecules, and their Resistance to Displacement Constituting Hardness..............30
ART. VIII.—The Bogus Diploma Traffic..............34
ART. IX.—The West Virginia Dental Law..............36
ART. X.—Is Mercury Absorbed by the Skin?..............37
ART. XI.—The Structure of Metals..............38

### EDITORIAL, ETC.

Female Doctors..............40
Wanted..............40

### BIBLIOGRAPHICAL.

Plastics and Plastic Fillings. By J. Foster Flagg, D. D. S..............41
The Illustrated Scientific News..............42
The Ohio State Journal of Dental Science..............42

### MONTHLY SUMMARY.

Holland's Dental Law..............43
Extraction of Teeth followed by Insanity..............45
To Prevent the Clouding of Mirrors by Moisture..............46
A Case of Fracture of the Teeth Producing Severe Symptoms..............47
Alcohol a Stimulant or Narcotic..............48

